# Osteoporosis treatment indications following fracture: identifying relevant fracture sites for Fracture Liaison Services

**DOI:** 10.1007/s11657-026-01690-0

**Published:** 2026-03-21

**Authors:** Mattias Lorentzon, Christine Florberger, Javier Merina, Eric Bertholds, Henrik Litsne, Kristian F. Axelsson

**Affiliations:** 1 Sahlgrenska Osteoporosis Center, Department of Internal Medicine and Clinical Nutrition, Institute of Medicine, Sahlgrenska Academy, University of Gothenburg, Gothenburg, Sweden; 2 Department of Internal Medicine, Geriatrics, and Emergency Medicine, Sahlgrenska University Hospital Mölndal, Region Västra Götaland, Mölndal, Sweden; 3 Osteoporosis Center, Skaraborg Hospital, Skövde, Sweden; 4 Närhälsan Norrmalm Health Centre, Region Västra Götaland, Skövde, Sweden

**Keywords:** Fracture Liaison Service, Major osteoporotic fracture, Prevention

## Abstract

***Summary*:**

Osteoporotic fractures are associated with morbidity, mortality and high healthcare costs. Fracture Liaison Services (FLS) prevent subsequent osteoporotic fractures but are traditionally limited to include major osteoporotic fractures (MOF). When the FLS at Skaraborg Hospital in Skövde, Sweden included both MOF and non-MOF, treatment indication was present for 51% (Number Needed to Screen (NNS) 1.41) and 71% (NNS 1.95), respectively. High treatment indication rates and low NNS were observed both in patients with MOF and non-MOF, suggesting that all fracture patients should be included in FLSs.

**Background:**

Fracture Liaison Services (FLS) are coordinator-based, multidisciplinary programmes that provide systematic secondary prevention of fragility fractures. Many FLS programmes have been limited to major osteoporotic fractures (MOF, i.e. vertebrae, hip, proximal humerus, wrist and pelvis), but it is unclear whether patients with other types of fractures have similar risk profiles, as defined by low bone mineral density (BMD) and present clinical risk factors (CRF).

**Objective:**

To compare key characteristics related to fracture risk (e.g. FRAX and BMD) and eligibility for osteoporosis treatment between patients with a recent non-MOF and those with recent MOF after inclusion in an FLS at Skaraborg Hospital in Skövde, Sweden.

**Methods:**

Patients 50 years and older with a BMD measurement between December 2023 and May 2024, with a recent fracture were included (*N* = 705). Data on age, sex, CRFs, FRAX score, BMD, trabecular bone score, vertebral fracture assessment (VFA), and physician-issued assessment of osteoporosis treatment indication from the FLS evaluation were collected. Differences were analyzed using *t*-tests, chi-square tests, and expressed as standardized mean differences. The odds ratio (OR) for osteoporosis treatment indication (yes/no) was calculated using logistic regression for non-MOF vs. MOF, with adjustment for incremental number of confounders.

**Results:**

There were high rates of osteoporosis treatment indication in both non-MOF (51%) and MOF (71%) patients, and low numbers needed to screen (NNS) to identify one patient with osteoporosis treatment indication in both the non-MOF (1.95) and MOF groups (1.41). When comparing non-MOF and MOF within the subgroup of patients with osteoporosis treatment indication, BMD and risk profiles were similar.

**Conclusion:**

The proportions of patients with osteoporosis treatment indications were high regardless of fracture site category, indicating that patients with both recent non-MOF and MOF should be included in FLS programmes.

**Supplementary Information:**

The online version contains supplementary material available at 10.1007/s11657-026-01690-0.

## Introduction

Fractures lead to a comprehensive increase in morbidity, mortality and healthcare costs, which with an ageing population will be a growing challenge globally [1, 2]. For patients over 50 years, the lifetime risk for osteoporotic fractures for women is 50% and 20% for men [3]. Swedish women have the highest incidence of hip fractures in the world, and the cost of osteoporotic fractures reached 2.3 billion Euro, approximately 4.3% of the total Swedish healthcare budget in 2019 [4]. The risk of subsequent fracture is the highest, increased up to 5 times the first 2 years after the index fracture, with a decline in risk in the following period but remains substantially elevated for over 10 years [5, 6].

Effective osteoporosis medications that increase bone mineral density (BMD) and reduce the risk of vertebral and nonvertebral fractures are available for decades [7, 8]. Although the evidence base is solid and the cost for most osteoporosis medications is very low, only a minority of patients receive adequate treatment [8–10]. Structured secondary prevention programmes—known as Fracture Liaison Services (FLS)—have been implemented to identify patients with fractures, assess them for osteoporosis and initiate appropriate treatment to prevent subsequent osteoporotic fractures [11, 12]. However, there is still no clear consensus regarding which fracture sites should be included [13].

Although most guidelines consistently recommend that FLS programmes include all fractures, many programmes are restricted to include hip and vertebra fractures or only major osteoporotic fractures (MOF; hip, vertebra, wrist, upper arm) [13–15]. However, the association of MOF with low BMD, osteoporosis and increased risk of subsequent fractures [16] does not imply that non-MOF are not similarly associated. Low BMD has been linked to fractures at most sites [16]. Furthermore, the commonly used term *fragility fracture* implies that only low-energy fractures should be included, which is not consistent with studies reporting that both high- and low-energy trauma fractures show comparable associations with low BMD and future fracture risk [17, 18]. Recent studies have demonstrated that most index fracture sites—not only the traditional MOF sites—are associated with an increased risk of subsequent fractures [19, 20], indicating that both MOF and non-MOF index fractures confer an elevated risk of future fractures.

The aim of this study was to use real-world data from an FLS in Sweden to compare key characteristics, BMD, clinical risk factors and osteoporosis treatment eligibility amongst FLS patients aged 50 years and older with non-MOF versus MOF fractures, in order to inform decisions on patient inclusion criteria for FLS programmes.

## Methods

### Study participants

In this retrospective cohort study, data was collected at the Osteoporosis clinic in Skaraborg Hospital in Skövde, Sweden, serving approximately 265,000 inhabitants. Patients over 50 years with any recent fracture were included in the department’s FLS regardless of trauma energy level, but excluding malignant fractures or fractures of the head, foot and hand. Patients with a dual X-ray absorptiometry (DXA) measurement between December 2023 and May 2024 were included in the study (Table S1). ICD-10 codes were used to categorize the recent fracture as either MOF (defined as fractures of the hip, vertebrae, proximal humerus, wrist or pelvis) or non-MOF, which included all other fracture sites (Table S2). Pelvic fractures were included in the MOF group to avoid missing vertebral fractures classified using ICD-10 combination codes used for vertebral and/or pelvic fractures. The study was approved by the Swedish Ethical Review Authority (2024–05455-01).

### Variables

Age and sex were derived from the patient’s personal identification number. The other clinical risk factors (CRF) included in the FRAX tool were collected from questionnaires that the patients completed at the hospital’s osteoporosis clinic (Table S3). The FRAX variables included parental hip fracture, smoking, oral glucocorticoid exposure (≥ 5 mg of prednisolone, for more than 3 months), rheumatoid arthritis, secondary osteoporosis and alcohol intake (≥ 3 standard alcohol units/day). Weight and height were measured at the osteoporosis clinic using standardized equipment. BMD was measured by International Osteoporosis Foundation/International Society for Clinical Densitometry certified operators using dual x-ray absorptiometry (DXA, Hologic Horizon A. Sites measured were femoral neck, total hip and lumbar spine (L1–L4). The occurrence of vertebral fractures was diagnosed with a lateral projection of the spine, vertebral fracture assessment (VFA), and in undetermined cases, additional X-ray examinations were ordered. Trabecular bone score (TBS) was calculated by DXA machine software, where a value below 1.2 was considered a risk indicator for increased fracture risk. Information on prevalent osteoporosis treatment and fractures was collected from medical records and the hospital X-ray archive, respectively.

Four different certified physicians (Table S4) reviewed all the available data and assessed osteoporosis treatment indication which were categorized as ‘No treatment’, ‘Inconclusive’, ‘Parenteral treatment’ or ‘Osteoanabolic treatment’. The physicians based their assessment on the Swedish national guidelines criteria for treatment eligibility, which include (i) previous hip or spine fracture, (ii) other fracture and *T*-score less than −1.0 SD and a FRAX MOF probability of 20% or more, (iii) a *T*-score ≤ −2.5 SD and a FRAX-score ≥ 20%, (iv) oral glucocorticoid treatment (> 5 mg prednisolone equivalents daily > 3 months) [21]. ‘Inconclusive’ referred to patients with an identified intermediary risk of future fracture, and where the decision of treatment was delegated to the primary care physician who was recommended to see the patient to provide a final assessment of comorbidities and patient motivation, further guiding the treatment choice.

### Statistical analyses

Key characteristics for the groups non-MOF and MOF were described with numbers and percentages for categorical values and mean (SD) for continuous variables. Group differences were investigated using chi-square test for categorical variables and *t*-test for continuous variables as well as with standardized mean differences (SMD). The categorical variables were sex, heredity, smoking, glucocorticoids, rheumatoid arthritis, secondary osteoporosis, alcohol, previous fracture, multiple fractures, VFA, previous DXA examination, previous osteoporosis treatment and treatment indications. The continuous variables were age, weight, height, body mass index (BMI), *T*-score femoral neck, *T*-score total hip, *T*-score lumbar spine, months since fracture, TBS and FRAX MOF probability. A *p*-value below 0.05 was considered significant.

First, all patients were analyzed, and treatment indication was compared between the non-MOF and MOF groups. Numbers needed to screen (NNS) in each group were obtained by inverting the osteoporosis indication rate. Second, patients with inconclusive treatment indication were excluded and logistic regression was applied to obtain odds ratios (OR 95% CI) for treatment indication (yes or no) for non-MOF vs. MOF with gradually adding adjustment variables (age, sex, femoral neck BMD, TBS, previous fracture history, vertebral fracture, FRAX MOF probability and assessing physician). Third, in a subgroup, only patients with treatment indication were analyzed, and differences in key characteristics, CRFs and osteoanabolic treatment were compared. SPSS version 30.0.0.0 (172) and R Studio (2023.03.0 + 386) with R version 4.2.2 (2022-10−31) were used for all analyses. *p*-values < 0.05 were considered significant.

## Results

### Study population

The study included a total of 705 fracture patients with available DXA scans between October 2023 and May 2024, assessed in Skövde as part of the regional FLS covering Skaraborg, Sweden (Table S5). The mean patient age was 67.3 years and 490 (69.5%) were women. The site with the lowest *T*-score was used in the clinical assessment and was most commonly the femoral neck (42.0%) followed by the lumbar spine (33.8%) (Table S6). Vertebral Fracture Assessment was performed per routine, and 127 (18.0%) patients had one or more vertebral fractures (VF) and 58 (8.1%) had two or more VFs (Table S7). There were 267 (37.9%) patients included in the FLS due to a non-MOF and 438 (62.1%) due to a MOF. The non-MOF group had significantly fewer women, higher mean weight, greater mean height, a longer duration since their fracture, higher BMD, lower prevalence of VF, lower FRAX MOF 10-year probability, fewer previous DXA examinations and a lower proportion of previous osteoporosis treatment (Table 1).

**Table 1:** Baseline characteristics for all patients (*N* = 705) and according to non-MOF vs MOF

Variable	All	MOF	Non-MOF	*p*-value	SMD	Missing
	*N* = 705	*N* = 438	*N* = 267			Total (*n*)
Age, years, mean (SD)	67.3 (9.4)	67.9 (9.3)	66.4 (9.6)	0.03	0.16	0
Female sex, *n* (%)	490 (69.5)	334 (76.3)	156 (58.4)	< 0.001	0.39	0
Weight, kg, mean (SD)	78.4 (17.0)	76.7 (16.7)	81.3 (17.1)	0.001	0.27	1
Height, cm, mean (SD)	167.6 (9.5)	166.2 (9.0)	170.0 (9.7)	< 0.001	0.41	1
BMI, kg/m^2^, mean (SD)	27.9 (5.5)	27.8 (5.7)	28.1 (5.1)	0.50	0.05	1
Parental hip fracture, *n* (%)	124 (17.7)	77 (17.7)	47 (17.7)	1.00	0	4
Smoking, *n* (%)	78 (11.1)	47 (10.8)	31 (11.7)	0.82	0.03	3
Glucocorticoids, *n* (%)	64 (9.1)	41 (9.4)	23 (8.7)	0.85	0.03	4
Rheumatoid arthritis, *n* (%)	27 (3.9)	13 (3.0)	14 (5.3)	0.19	0.12	5
Secondary osteoporosis, *n* (%)	201 (28.6)	121 (27.8)	80 (30.0)	0.60	0.05	3
Alcohol, *n* (%)	13 (1.9)	9 (2.1)	4 (1.5)	0.81	0.04	5
Recent fracture, *n* (%)	705 (100)	438 (100)	267 (100)	1.00	0	0
≥ 2 fx at recent fx event, *n* (%)	131 (18.6)	76 (17.4)	55 (20.6)	0.33	0.08	0
Months since fracture(s)*, mean (SD)	1.98 (1.16)	1.90 (1.11)	2.12 (1.24)	0.01	0.19	0
Older fracture**, *n* (%)	306 (43.4)	181 (41.3)	125 (46.8)	0.18	0.11	0
*T*-score femoral neck, mean (SD)	− 1.84 (0.90)	− 1.95 (0.83)	− 1.66 (0.99)	< 0.001	0.31	14
*T*-score total hip, mean (SD)	− 1.00 (0.95)	− 1.10 (0.88)	− 0.85 (1.03)	0.001	0.26	14
*T*-score lumbar spine, mean (SD)	− 1.47 (1.34)	− 1.61 (1.29)	− 1.25 (1.41)	0.001	0.27	60
Vertebral fracture (VFA), *n* (%)	127 (18.0)	100 (22.8)	27 (10.1)	< 0.001	0.35	0
FRAX***, mean (SD)	21.4 (12.7)	22.8 (12.5)	19.4 (12.8)	0.001	0.27	96
TBS, mean (SD)	1.28 (0.11)	1.27 (0.11)	1.29 (0.12)	0.05	0.16	47
Previous DXA examination, *n* (%)	103 (14.6)	70 (16.0)	33 (12.4)	0.23	0.10	0
Previous osteoporosis treatment, *n* (%)	70 (9.9)	51 (11.6)	19 (7.1)	0.07	0.16	0

Baseline characteristics and comparison. *t*-test and chi-square test were used to compare the non-MOF group with the MOF group

*MOF* major osteoporotic fracture, *SMD* standardized mean difference, *SD* standardized difference, *Fx* fracture, *TBS* trabecular bone score, *FRAX*-definitions were used for parental hip fracture, smoking, glucocorticoids, rheumatoid arthritis, secondary osteoporosis and alcohol intake

*Time between fracture date and examination date

**Patients with a prior fracture before the fracture that qualified the patient for the FLS

***FRAX MOF 10-year probability

### High rates of treatment indication in both non-MOF and MOF

**Fig. 1:**
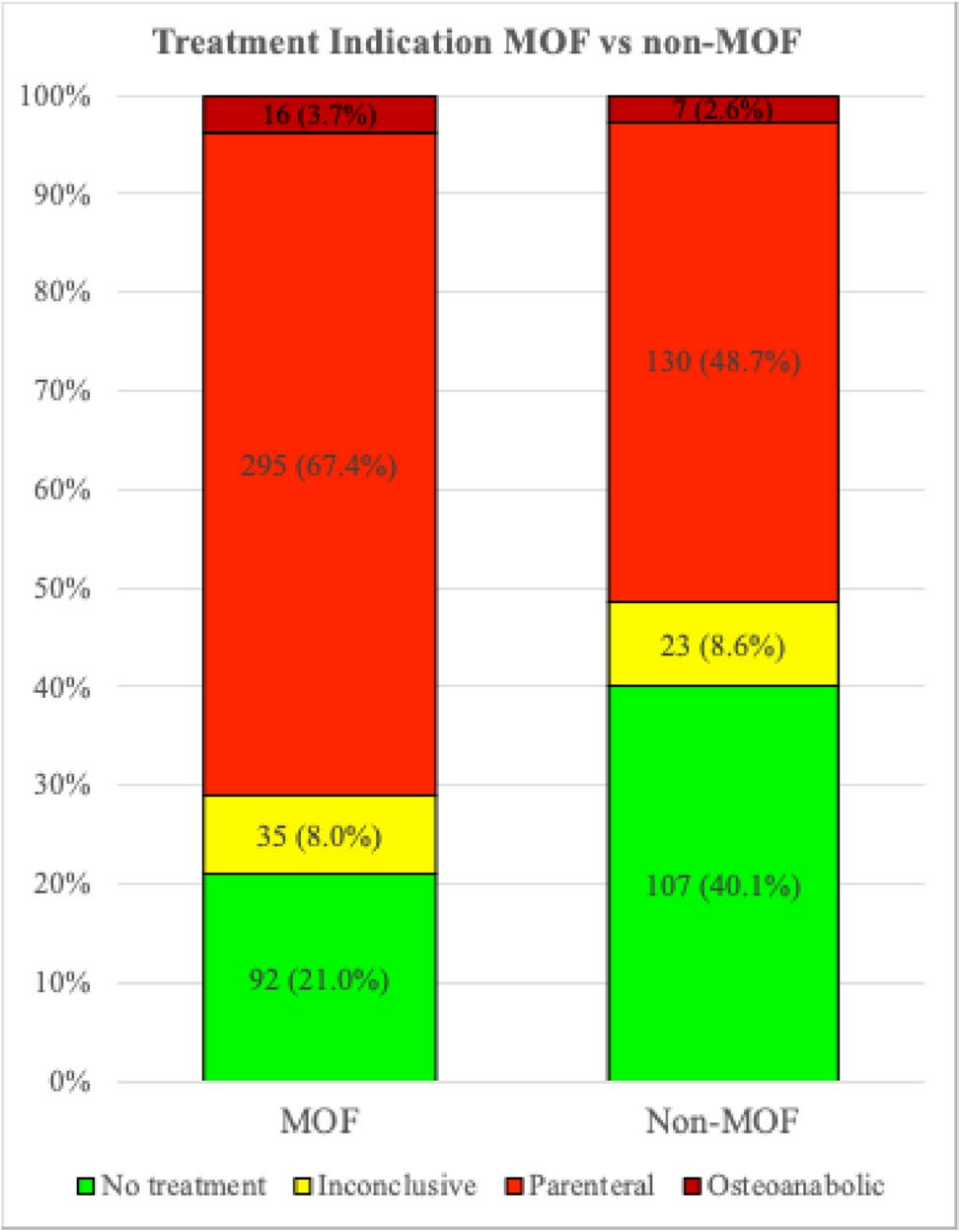
Osteoporosis treatment indication for all patients (*N* = 705) according to non-MOF vs MOF. Comparison of treatment indication categorized as no treatment, inconclusive, parenteral and anabolic for non-MOF vs MOF (*p* < 0.001, standardized mean difference 0.44). Inconclusive refers to patients with an identified intermediary risk of future fracture, and where the decision of treatment was delegated to the primary care physician who meets the patient and makes a final assessment of comorbidities and motivation. Parenteral included both zoledronic acid and denosumab, both first introduction and renewals (see Table S8 for further details)

Whilst there was a significant difference (*p* < 0.001) between the proportion of patients with treatment indication (parenteral or osteoanabolic), both groups had high rates, 51.3% in the non-MOF group and 71.1% in the MOF group, corresponding to NNS of 1.95 and 1.41, respectively (Fig. 1, Table S8). There were similar proportions of patients with treatment indication regardless of non-MOF fracture type, including fractures of the elbow, clavicle, rib, knee and ankle (Table S9).

### Association between treatment indication and index fracture category

**Fig. 2:**
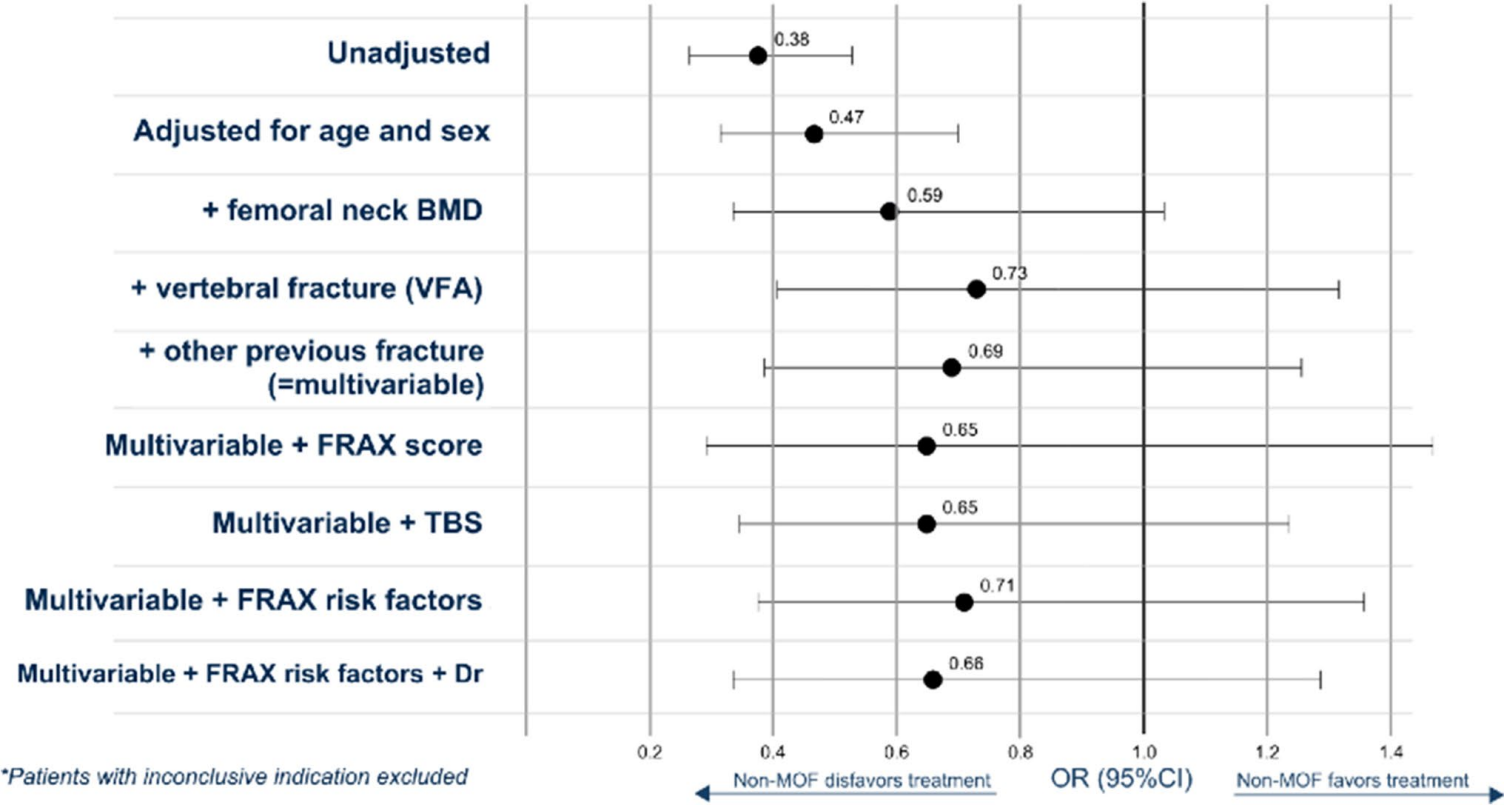
Odds ratios (OR) for osteoporosis treatment indication. After excluding 59 cases (8.4%) with inconclusive treatment indication, the non-MOF group was compared with MOF in an unadjusted logistic regression with increasing degrees of adjustment. The odds ratios including confidence intervals are presented here and in Table S10

After excluding cases with inconclusive treatment indication, the non-MOF group was compared with the MOF group using an unadjusted logistic regression model (Fig. 2, Table S10), revealing a 62% lower chance of receiving a treatment recommendation (OR 0.38, 95% Confidence Interval (CI) 0.27–0.53, *p* < 0.001). When adjusting for age, sex and femoral neck BMD, the chance was attenuated and non-significant (OR 0.59 (0.34–1.03), *p* = 0.06). The chance decreased and remained insignificant with further adjustment variables included, e.g. VFA, other previous fracture, FRAX-score, TBS, FRAX CRFs, assessing physician (Table S10).

### Analysis of patients with osteoporosis treatment indication according to non-MOF and MOF category

Focusing solely on the patients who received osteoporosis treatment indication (*N* = 448) and comparing non-MOF patients with MOF revealed similar characteristics for most variables (Table 2). There were no significant differences in non-MOF vs. MOF in mean age (69.8 vs 70.2), FRAX (28.4 vs 27.5), *T*-scores of the femoral neck (−2.3 vs −2.2), total hip (−1.5 vs −1.4), and lumbar spine (−2.0 vs −2.0). Women were significantly less common in the non-MOF group (67.9% vs 80.4%) than in the MOF group, and the proportion with vertebral fracture (16.8% vs 28.6%) was significantly lower. Patients in the non-MOF group had a higher prevalence of rheumatoid arthritis (8.1% vs 2.3%), secondary osteoporosis (38.0% vs 28.2%), and older fracture (56.9% vs 44.4%) than patients in the MOF group. In both the non-MOF and MOF groups, most patients were recommended antiresorptive parenteral treatment (94.9%) and the remaining (5.1%) were recommended osteoanabolic treatment.

**Table 2:** Baseline characteristics for patients with osteoporosis treatment indication (*N* = 448) according to non-MOF vs MOF. All patients (*N* = 705) included for reference

Variable	All	MOF	Non-MOF	*p*-value	SMD	Missing
	*N* = 705	*N* = 311	*N* = 137			Total (*n*)
Age, years, mean (SD)	67.3 (9.4)	70.2 (8.7)	69.8 (8.6)	0.62	0.05	0
Female sex, *n* (%)	490 (69.5)	250 (80.4)	93 (67.9)	0.006	0.29	0
Weight, kg, mean (SD)	78.4 (17.0)	74.1 (15.2)	75.7 (15.4)	0.29	0.11	1
Height, cm, mean (SD)	167.6 (9.5)	165.1 (8.6)	166.7 (9.4)	0.08	0.18	1
BMI, kg/m^2^, mean (SD)	27.9 (5.5)	27.2 (5.5)	27.2 (5.1)	0.96	0.01	1
Parental hip fracture, *n* (%)	124 (17.7)	64 (20.7)	36 (26.7)	0.21	0.14	4
Smoking, *n* (%)	78 (11.1)	32 (10.4)	18 (13.1)	0.49	0.09	3
Glucocorticoids, *n* (%)	64 (9.1)	37 (12.0)	21 (15.4)	0.40	0.10	4
Rheumatoid arthritis, *n* (%)	27 (3.9)	7 (2.3)	11 (8.1)	0.009	0.27	5
Secondary osteoporosis, *n* (%)	201 (28.6)	87 (28.2)	52 (38.0)	0.05	0.21	3
Alcohol, *n* (%)	13 (1.9)	7 (2.3)	4 (2.9)	0.93	0.04	5
Recent fracture, *n* (%)	705 (100)	311 (100)	137 (100)	1.00	0	0
≥ 2 fx at recent fx event, *n* (%)	131 (18.6)	47 (15.1)	29 (21.2)	0.15	0.16	0
Months since fracture(s)*, mean (SD)	1.98 (1.16)	1.90 (1.18)	2.26 (1.40)	0.004	0.28	0
Older fracture**, *n* (%)	306 (43.4)	138 (44.4)	78 (56.9)	0.02	0.25	0
*T*-score femoral neck, mean (SD)	− 1.84 (0.90)	− 2.22 (0.70)	− 2.32 (0.69)	0.17	0.14	14
*T*-score total hip, mean (SD)	− 1.00 (0.95)	− 1.38 (0.79)	− 1.49 (0.86)	0.19	0.14	14
*T*-score lumbar spine, mean (SD)	− 1.47 (1.34)	− 1.96 (1.17)	− 1.95 (1.14)	0.94	0.01	60
Vertebral fracture (VFA), *n* (%)	127 (18.0)	89 (28.6)	23 (16.8)	0.01	0.29	0
FRAX***, mean (SD)	21.43 (12.71)	27.51 (12.12)	28.43 (13.13)	0.52	0.07	96
TBS, mean (SD)	1.28 (0.11)	1.26 (0.10)	1.25 (0.10)	0.79	0.03	47
Previous DXA examination, *n* (%)	103 (14.6)	63 (20.3)	27 (19.7)	1.00	0.01	0
Previous osteoporosis treatment, *n* (%)	70 (9.9)	48 (15.4)	18 (13.1)	0.63	0.07	0
Treatment recommendation, *n* (%)				1.00	0	0
Parenteral medications	425 (60.3)	295 (94.9)	130 (94.9)			
Osteoanabolic medications	23 (3.3)	16 (5.1)	7 (5.1)			

Baseline characteristics and comparison non-MOF vs MOF in patients with osteoporosis treatment indication (*N* = 448, parenteral or anabolic). *t*-test and chi-square test were used to compare the non-MOF group with the MOF group

*MOF* major osteoporotic fracture, *SMD* standardized mean difference, *SD* standardized difference, *Fx* fracture, *TBS* trabecular bone score. *FRAX*-definitions were used for parental hip fracture, smoking, glucocorticoids, rheumatoid arthritis, secondary osteoporosis and alcohol intake

*Time between fracture date and examination date

**Patients with a prior fracture before the fracture that qualified the patient for the FLS

***FRAX MOF 10-year probability

### Comparison of treatment groups for all patients

Comparing the characteristics of the different treatment indication groups, no treatment (*N* = 199), inconclusive (*N* = 58), parenteral (*N* = 425) and osteoanabolic (*N* = 23) demonstrated significant differences between the groups (Table S11). As expected, patients with indication for osteoanabolic treatment had lower BMD and TBS, higher FRAX 10-year probability than those with indication for parenteral treatment or those with inconclusive indication (Table S11).

## Discussion

A substantial proportion of patients—51% in the non-MOF group and 71% in the MOF group—were recommended osteoporosis medication during the FLS assessment. These figures correspond to very low numbers needed to screen of 1.95 and 1.41, respectively, indicating that only a small number of patients with fractures, irrespective of fracture site, require screening to identify those with a treatment indication. As no consensus currently exists regarding which fracture sites should be included in FLS programmes—with many centres limiting inclusion to MOF sites—the findings of this study provide important evidence supporting the inclusion of additional fracture sites, given the high proportion of patients identified as eligible for treatment [13].

Overall, patients with MOF index fracture site had lower BMD and higher FRAX probability than patients with fractures at non-MOF sites, but when these comparisons were restricted to patients with treatment indication, those in the non-MOF group more frequently had multiple fractures (*p* = 0.15), another older fracture (*p* = 0.02) and similar BMD on average (*p* > 0.05), which indicates that a greater risk profile might have been required for being treatment eligible in the non-MOF group. Additional analyses revealed that index MOF was significantly associated with increased odds ratio of being treatment eligible, but when adjusting for BMD, VFA and additional risk factors, this association was no longer significant, indicating that index fracture site per se does not dictate treatment eligibility alone.

Previous large national register studies from Sweden have demonstrated an increased risk of subsequent any, hip and vertebral fracture regardless of index fracture site, but analyses did not account for BMD and lacked information about several self-reported clinical risk factors [19, 20]. The present study incorporated BMD, TBS, VFA and relevant self-reported clinical risk factors, enabling a state-of-the-art fracture risk assessment and providing comprehensive data to inform decisions on osteoporosis medication indication. Based on this detailed risk profiling, a high proportion of treatment-eligible patients was identified in both the non-MOF and MOF groups, supporting the clinical relevance of the increased fracture risk identified in patients with recent non-MOF in the Swedish register cohorts. Although our study did not include controls without fracture, BMD *T*-scores were on average in the osteopenic range in both the non-MOF and MOF groups, in agreement with the previously reported low BMD observed in patients with fractures at most sites [16].

In a large Swedish FLS study encompassing four hospitals with historic controls, Axelsson et al. observed an 18% reduction in the rate of subsequent fractures in those evaluated during the FLS period, compared to the pre-FLS period, but the FLS only included patients with MOF [11]. Given the high proportion of treatment eligible patients in the non-MOF group, widening of such an FLS to also include non-MOF would increase the proportion of fracture patients evaluated and treated, possibly reducing the rates of subsequent fractures even further.

These findings are based on data from a regional hospital and the assessment by certified physicians using treatment thresholds derived from the Swedish national guidelines, which might limit the generalizability to other settings with different fracture risks and treatment thresholds. It should be acknowledged that in most clinical settings, including all fracture sites, not just MOF, would require additional use of DXA. Although costs would increase, the results from this study indicate that there is no reason to favour patients with MOF over non-MOF.

The extensive characterization of fracture patients with inclusion of clinical risk factors, TBS, VFA, BMD measurements and detailed treatment recommendations constitutes a major strength of the study. Furthermore, the analysis was controlled for assessing physician and did not reveal signs of bias. Limitations of the study should be acknowledged. Although the study was sufficiently powered, the short recruitment time of 6 months did not allow investigating seasonal variations and extended subgroup analysis according to index fracture site. Importantly, the study was not blinded, i.e. the assessing physician had knowledge of the fracture type, which might have affected the assessment of treatment indication. Also, whilst the Swedish guidelines were used by all physicians, there is a risk of physician-dependent bias. However, physicians held regular meetings to harmonize their assessments, and adjustment for the assessing physician did not materially alter the observed results. Trauma severity was not considered in the inclusion of fracture patients or in the presented analysis. However, several studies have found similar associations between previous fracture, regardless of trauma type, and risk of subsequent fracture [17, 18]. In contrast to the most common MOF definition used, we also included pelvic fractures. This is a limitation, but likely of minor consequence given the similar characteristics between the MOF and non-MOF groups and the small number of pelvic fractures (34/438 = 7.8%). 

This study demonstrated that a large proportion of patients—51% in the non-MOF group and 71% in the MOF group—met the criteria for osteoporosis treatment during the FLS evaluation. The resulting low numbers needed to screen (1.95 and 1.41, respectively) highlight the efficiency of comprehensive FLS assessments in identifying patients requiring pharmacological intervention, irrespective of index fracture site.

## Supplementary Information

Below is the link to the electronic supplementary material.

ESM 1 (DOCX 85.4 KB)

## Data Availability

Data cannot be made publicly available for ethical and legal reasons. Such information is subject to legal restrictions according to national legislation. Specifically, in Sweden, confidentiality regarding personal information in studies is regulated in the Public Access to Information and Secrecy Act (SFS 2009:400). The data underlying the results of this study might be made available upon request, after an assessment of confidentiality. There is thus a possibility to apply to get access to certain public documents that an authority holds. In this case, the University of Gothenburg is the specific authority that is responsible for the integrity of the documents with research data. Questions regarding such issues can be directed to the head of the Institute of Medicine, Sahlgrenska Academy, University of Gothenburg, Gothenburg, Sweden. Contact information can be obtained from medicin@gu.se.
