# Supplementary material for: Osteoporosis treatment indications following fracture: identifying relevant fracture sites for Fracture Liaison Services
Source: Arch Osteoporos. 2026 Mar 21;21(1):54. doi: 10.1007/s11657-026-01690-0 (PMC13005837; doi:10.1007/s11657-026-01690-0)
Supplement: Supplementary file 1 — (DOCX 85.4 KB) [file 11657_2026_1690_MOESM1_ESM.docx]

**Supplementary Appendix**

To

**Osteoporosis Treatment Indications Following Fracture:**

**Identifying Relevant Fracture Sites for Fracture Liaison Services**

By

Mattias Lorentzon, Christine Florberger,

Javier Merina, Eric Bertholds, Henrik Litsne,

Kristian F. Axelsson

**Table of Contents**

[Table S1. Month of DXA examination 3](#_Toc211934801)

[Table S2A. Definition of non-MOF (N=267) per fracture sites using ICD-10 codes 4](#_Toc211934802)

[Table S2B. Definition of MOF (N=438) per fracture sites using ICD-10 codes 5](#_Toc211934803)

[Table S3 Patient questionnaire 6](#_Toc211934804)

[Table S4. Assessing Physician 6](#_Toc211934805)

[Table S5. Municipality of patient residence 7](#_Toc211934806)

[Table S6. DXA site with lowest T-score used in clinical assessment 7](#_Toc211934807)

[Table S7. VFA assessment 8](#_Toc211934808)

[Table S8. Treatment recommendation – all patients (N=705), non-MOF vs MOF 8](#_Toc211934809)

[Table S9. Baseline characteristics, all patients (N=705) non-MOF split per subgroups vs. MOF 9](#_Toc211934810)

[Table S10. Odds ratios for treatment recommendation, non-MOF vs. MOF 10](#_Toc211934811)

[Table S11. Baseline characteristics, all patients per treatment recommendation 11](#_Toc211934812)

## Table S1. Month of DXA examination

| Oct, 2023 | 61 |
| --- | --- |
| Nov, 2023 | 81 |
| Dec, 2023 | 53 |
| Jan, 2024 | 94 |
| Feb, 2024 | 115 |
| Mar, 2024 | 116 |
| Apr, 2024 | 117 |
| May, 2024 | 68 |
|  |  |
|  | 705 |

## Table S2A. Definition of non-MOF (N=267) per fracture sites using ICD-10 codes

| **Non-Major Osteoporotic Fracture (non-MOF)** |  |  |
| --- | --- | --- |
|  |  |  |
| Elbow | 45 | 6.4% |
| S424 lower end of humerus | 9 | 1.3% |
| S520 upper end of ulna | 18 | 2.6% |
| S521 upper end of radius | 18 | 2.6% |
|  |  |  |
| Clavicle | 16 | 2.3% |
| S420 clavicle | 16 | 2.3% |
|  |  |  |
| Rib | 36 | 5.1% |
| S223 rib | 18 | 2.6% |
| S224 Multiple fractures of ribs | 18 | 2.6% |
|  |  |  |
| Knee | 46 | 6.5% |
| S724 lower end of femur | 2 | 0.3% |
| S820 patella | 13 | 1.8% |
| S821 upper end of tibia | 31 | 4.4% |
|  |  |  |
| Ankle | 92 | 13.0% |
| S823 lower end of tibia | 2 | 0.3% |
| S825 medial malleolus | 6 | 0.9% |
| S826 lateral malleolus | 43 | 6.1% |
| S828 other parts of lower leg | 41 | 5.8% |
|  |  |  |
| Other | 32 | 4.5% |
| S222 sternum | 1 | 0.1% |
| S225 flail chest | 1 | 0.1% |
| S421 scapula | 5 | 0.7% |
| S423 shaft of humerus | 2 | 0.3% |
| S428 other parts of shoulder and upper arm | 1 | 0.1% |
| S429 shoulder girdle, part unspecified | 1 | 0.1% |
| S522 shaft of ulna | 3 | 0.4% |
| S523 shaft of radius | 2 | 0.3% |
| S528 other parts of forearm | 2 | 0.3% |
| S723 shaft of femur | 1 | 0.1% |
| S824 fibula alone | 12 | 1.7% |
| S827 multiple fractures of lower leg | 1 | 0.1% |
|  |  |  |
| Total non-MOF | 267 | 37.9% |

## Table S2B. Definition of MOF (N=438) per fracture sites using ICD-10 codes

| **Major Osteoporotic Fracture (MOF)** |  |  |
| --- | --- | --- |
|  |  |  |
| Wrist | 225 | 31.9% |
| S525 lower end of radius | 218 | 30.9% |
| S526 lower end of both ulna and radius | 7 | 1.0% |
|  |  |  |
| Proximal humerus | 97 | 13.8% |
| S422 upper end of humerus | 97 | 13.8% |
|  |  |  |
| Vertebral | 54 | 7.7% |
| M485 collapsed vertebra, not elsewhere classified | 3 | 0.4% |
| S120 first cervical vertebra | 1 | 0.1% |
| S121 second cervical vertebra | 1 | 0.1% |
| S122 other specified cervical vertebra | 4 | 0.6% |
| S220 thoracic vertebra | 19 | 2.7% |
| S320 lumbar vertebra | 25 | 3.5% |
| T089 spine, level unspecified | 1 | 0.1% |
|  |  |  |
| Vertebral and/or pelvis | 11 | 1.6% |
| S32 lumbar spine and pelvis | 2 | 0.3% |
| S327 multiple fractures of lumbar spine and pelvis | 8 | 1.1% |
| S328 other and unspecified parts of lumbar spine and pelvis | 1 | 0.1% |
|  |  |  |
| Pelvis | 23 | 3.3% |
| S321 sacrum | 4 | 0.6% |
| S323 ilium | 1 | 0.1% |
| S325 pubis | 18 | 2.6% |
|  |  |  |
| Hip | 28 | 4.0% |
| S720 neck of femur | 15 | 2.1% |
| S721 pertrochanteric fracture | 13 | 1.8% |
| S722 subtrochanteric fracture | 0 | 0.0% |
|  |  |  |
| **Total MOF** | **438** | **62.1%** |

## Table S3 Patient questionnaire

1. Have you had a fracture after 40 years of age?
2. Have your parents had a fracture of the hip?
3. Do your parents/ siblings have osteoporosis?
4. Do you smoke (if yes, how many cigarettes per day)?
5. Have you received a glucocorticoid treatment (if yes: name, dosage and when)?
6. Have you reveived osteoporosis treatment (if yes: name, dosage and when)?
7. Please list your current medications.
8. Do you have Rheumatoid arthritis (RA)?
9. Do you have any of the following diseases/operations:

- Thyroid disease
- Renal failure
- Diabetes
- IBD
- Celiac disease
- Asthma/COPD
- Gastric bypass operation

1. Other chronic disease?
2. Do you consume more than three units' alcohol per day?
3. Your ethnicity
4. Have you fallen in the last 12 months?

For women:

1. Do you receive estrogen treatment?
2. Have you removed your ovaries?
3. Have your menstruation ended (if yes: when)?

## Table S4. Assessing Physician

| Dr 1 (EB) | 330 |
| --- | --- |
| Dr 2 (JM) | 299 |
| Dr 3 | 64 |
| Dr 4 | 12 |
|  |  |
|  | 705 |

## Table S5. Municipality of patient residence

| Skövde | 138 |
| --- | --- |
| Lidköping | 117 |
| Falköping | 86 |
| Mariestad | 75 |
| Skara | 47 |
| Götene | 41 |
| Töreboda | 37 |
| Tidaholm | 33 |
| Vara | 30 |
| Hjo | 28 |
| Tibro | 27 |
| Karlsborg | 19 |
| Gullspång | 15 |
| Essunga | 5 |
| Grästorp | 5 |
| Göteborg | 1 |
| Missing | 1 |
|  |  |
|  | 705 |

## Table S6. DXA site with lowest T-score used in clinical assessment

| Lumbar spine | 238 | 33.8% |
| --- | --- | --- |
| Femoral neck, both | 296 | 42.0% |
| Femoral neck, left | 58 | 8.2% |
| Femoral neck, right | 50 | 7.1% |
| Total hip, both | 17 | 2.4% |
| Total hip, left | 2 | 0.3% |
| Total hip, right | 4 | 0.6% |
| 1/3 Radius left | 29 | 4.1% |
| 1/3 Radius right | 6 | 0.9% |
| Missing | 5 | 0.7% |
|  |  |  |
|  | 705 | 100.0% |

## Table S7. VFA assessment

| **No fracture** | **578** | **82.0%** |
| --- | --- | --- |
| Negative VFA | 570 | 80.9% |
| Not examined | 8 | 1.1% |
|  |  |  |
| **Vertebral fracture (s)** | **127** | **18.0%** |
| most likely | 4 | 0.6% |
| 1 | 65 | 9.2% |
| 2 | 32 | 4.5% |
| 3 | 15 | 2.1% |
| 4 | 3 | 0.4% |
| 5 | 3 | 0.4% |
| 6 | 2 | 0.3% |
| 7 | 1 | 0.1% |
| 10 | 2 | 0.3% |
|  |  |  |
|  | 705 | 100.0% |

## Table S8. Treatment recommendation – all patients (N=705), non-MOF vs MOF

Treatment indication categorized as no treatment, inconclusive, parenteral and anabolic. Inconclusive’ referred to patients with an identified intermediary risk of future fracture, but where the decision of treatment was delegated to the primary care physician who meets the patient and makes a final assessment of comorbidities and motivation.

| **No treatment** | **198** | **28.1%** |
| --- | --- | --- |
| No indication | 197 | 27.9% |
| Paus recommended | 1 | 0.1% |
|  |  |  |
| **Inconclusive** | **59** | **8.4%** |
| Borderline | 57 | 8.1% |
| Referring doctor decides | 2 | 0.3% |
|  |  |  |
| **Parenteral treatment** | **425** | **60.3%** |
| Parenteral | 370 | 52.5% |
| Denosumab | 36 | 5.1% |
| Parenteral (continued) | 5 | 0.7% |
| Denosumab (continued) | 12 | 1.7% |
| Continued treatment | 2 | 0.3% |
|  |  |  |
| **Osteoanabolic** | **23** | **3.3%** |
|  |  |  |
| **Total** | **705** | **100.0%** |

## Table S9. Baseline characteristics, all patients (N=705) non-MOF split per subgroups vs. MOF

|  |  |  |  |  |  |  |  |
| --- | --- | --- | --- | --- | --- | --- | --- |
| Variable | MOF | Elbow | Clavicle | Rib | Knee | Ankle | Other |
|  | N=438 | N=45 | N=16 | N=36 | N=46 | N=92 | N=32 |
| Age, mean (SD) | 67.90 (9.29) | 66.80 (8.55) | 69.56 (10.71) | 68.00 (10.29) | 67.76 (10.36) | 64.32 (9.43) | 66.09 (8.61) |
| Sex, n (%) | 334 (76.3) | 28 (62.2) | 7 (43.8) | 11 (30.6) | 31 (67.4) | 55 (59.8) | 24 (75.0) |
| Weight, mean (SD) | 76.71 (16.68) | 80.64 (18.14) | 73.56 (15.38) | 80.33 (16.24) | 77.28 (15.46) | 86.54 (17.92) | 77.84 (13.87) |
| Height, mean (SD) | 166.17 (9.02) | 170.62 (10.28) | 170.38 (12.23) | 172.00 (9.48) | 169.11 (8.68) | 170.49 (9.46) | 166.41 (9.30) |
| BMI, mean (SD) | 27.78 (5.67) | 27.60 (5.03) | 25.15 (3.25) | 27.11 (4.76) | 26.98 (4.90) | 29.70 (5.45) | 28.14 (4.81) |
| Parent fractured hip, n (%) | 77 (17.7) | 7 (15.6) | 3 (18.8) | 10 (28.6) | 7 (15.6) | 13 (14.1) | 7 (21.9) |
| Smoking, n (%) | 47 (10.8) | 6 (13.3) | 2 (12.5) | 6 (17.1) | 2 (4.3) | 10 (10.9) | 5 (15.6) |
| Glucocorticoids, n (%) | 41 (9.4) | 2 (4.4) | 3 (18.8) | 4 (11.4) | 5 (11.1) | 7 (7.6) | 2 (6.2) |
| Rheumatoid arthritis, n (%) | 13 (3.0) | 3 (6.7) | 3 (18.8) | 1 (2.8) | 2 (4.4) | 3 (3.3) | 2 (6.2) |
| Secondary osteoporosis, n (%) | 121 (27.8) | 15 (33.3) | 4 (25.0) | 13 (36.1) | 8 (17.4) | 28 (30.4) | 12 (37.5) |
| Alcohol, n (%) | 9 (2.1) | 0 (0.0) | 0 (0.0) | 1 (2.8) | 1 (2.2) | 2 (2.2) | 0 (0.0) |
| Recent fracture, n (%) | 438 (100) | 45 (100) | 16 (100) | 36 (100) | 46 (100) | 92 (100) | 32 (100) |
| ≥2 fx at recent fx event, n (%) | 76 (17.4) | 6 (13.3) | 2 (12.5) | 27 (75.0) | 9 (19.6) | 6 (6.5) | 5 (15.6) |
| Months since fracture(s)*, mean (SD) | 1.90 (1.11) | 2.00 (0.93) | 2.18 (1.80) | 2.31 (1.55) | 2.15 (1.09) | 1.97 (0.87) | 2.44 (1.87) |
| Older fracture**, n (%) | 181 (41.3) | 24 (53.3) | 9 (56.2) | 18 (50.0) | 22 (47.8) | 38 (41.3) | 14 (43.8) |
| T-score femoral neck, mean (SD) | -1.95 (0.83) | -1.90 (1.08) | -1.91 (0.72) | -1.70 (1.07) | -1.83 (0.99) | -1.48 (0.96) | -1.43 (0.89) |
| T-score total hip, mean (SD) | -1.10 (0.88) | -1.23 (1.11) | -1.16 (0.89) | -0.80 (1.17) | -1.07 (0.92) | -0.62 (0.95) | -0.58 (0.96) |
| T-score lumbar spine, mean (SD) | -1.61 (1.29) | -1.71 (1.43) | -1.06 (1.68) | -1.12 (1.77) | -1.37 (1.13) | -0.96 (1.27) | -1.35 (1.44) |
| Vertebral fracture (VFA), n (%) | 100 (22.8) | 8 (17.8) | 0 (0.0) | 6 (16.7) | 3 (6.5) | 9 (9.8) | 1 (3.1) |
| FRAX***, mean (SD) | 22.78 (12.49) | 21.56 (12.07) | 22.27 (14.80) | 18.81 (13.23) | 22.02 (16.25) | 16.99 (10.97) | 18.46 (10.58) |
| TBS, mean (SD) | 1.27 (0.11) | 1.25 (0.11) | 1.31 (0.12) | 1.31 (0.11) | 1.31 (0.08) | 1.29 (0.14) | 1.30 (0.10) |
| Previous DXA examination, n (%) | 70 (16.0) | 5 (11.1) | 2 (12.5) | 6 (16.7) | 4 (8.7) | 12 (13.0) | 4 (12.5) |
| Previous osteoporosis treatment, n (%) | 51 (11.6) | 0 (0.0) | 1 (6.2) | 5 (13.9) | 4 (8.7) | 5 (5.4) | 4 (12.5) |
| Treatment recommendation, n (%) |  |  |  |  |  |  |  |
| No treatment | 92 (21.0) | 15 (33.3) | 4 (25.0) | 19 (52.8) | 16 (34.8) | 41 (44.6) | 12 (37.5) |
| Inconclusive | 35 (8.0) | 4 (8.9) | 4 (25.0) | 0 (0.0) | 4 (8.7) | 9 (9.8) | 2 (6.2) |
| Parenteral | 295 (67.4) | 21 (46.7) | 8 (50.0) | 17 (47.2) | 25 (54.3) | 41 (44.6) | 18 (56.2) |
| Osteoanabolic bone formation | 16 (3.7) | 5 (11.1) | 0 (0.0) | 0 (0.0) | 1 (2.2) | 1 (1.1) | 0 (0.0) |

Baseline characteristics and comparisons of MOF to detailalled non-MOF fracture group (elbow, clavicle, rib, knee, ankle, other). per treatment. T-test and chi-square test were used. SMD = Standardized mean difference. SD = Standardized Difference. Fx = Fracture. TBS = Trabecular bone score. * Time between fracture date and examination date. ** Patients with a prior fracture before the fracture that qualified the patient for the FLS. *** FRAX MOF 10-year probability

## Table S10. Odds ratios for treatment recommendation, non-MOF vs. MOF

|  |  |  |  |  |  |
| --- | --- | --- | --- | --- | --- |
| Model | Adjustment | MOF | Non-MOF |  |  |
|  |  | N=403 | N=244 |  |  |
|  |  |  | OR (95% CI) | p-value | #missing |
| Model 1 | Non-MOF vs. MOF, unadjusted | Ref. [1.0] | 0.38 (0.27-0.53) | <0.001 | 0 |
| Model 2 | Adjusted for age and sex | Ref. [1.0] | 0.47 (0.32-0.70) | <0.001 | 0 |
| Model 3 | + femoral neck BMD | Ref. [1.0] | 0.59 (0.34-1.03) | 0.06 | 14 |
| Model 4 | + vertebral fracture (VFA) | Ref. [1.0] | 0.73 (0.41-1.31) | 0.29 | 14 |
| Model 5 | + other previous fracture (= multivariable) | Ref. [1.0] | 0.69 (0.39-1.25) | 0.22 | 14 |
| Model 6 | Multivariable + FRAX* score | Ref. [1.0] | 0.65 (0.29-1.46) | 0.30 | 99 |
| Model 7 | Multivariable + TBS | Ref. [1.0] | 0.65 (0.35-1.23) | 0.19 | 54 |
| Model 8 | Multivariable + FRAX risk factors | Ref. [1.0] | 0.71 (0.38-1.35) | 0.30 | 21 |
| Model 9 | Multivariable + FRAX risk factors + Dr | Ref. [1.0] | 0.66 (0.34-1.28) | 0.22 | 21 |

Patient with inconclusive treatment recommendations were excluded (58), with remaining N=647 analyzed with logistic regression to obtain odds ratios (OR 95% CI) for treatment recommendation (yes or no) for non-MOF compared to MOF with gradually adding number of adjustment variables.

* FRAX MOF 10-year probability. Dr=assessing physician.

## Table S11. Baseline characteristics, all patients per treatment recommendation

|  |  |  |  |  |  |  |
| --- | --- | --- | --- | --- | --- | --- |
| Variable | No treatment | Inconclusive | Parenteral | Osteanabolic | p-value | SMD |
|  | N=199 | N=58 | N=425 | N=23 |  |  |
| Age, mean (SD) | 61.72 (8.61) | 65.09 (9.08) | 70.00 (8.67) | 71.74 (8.21) | <0.001 | 0.68 |
| Female sex, n (%) | 107 (53.8) | 40 (69.0) | 324 (76.2) | 19 (82.6) | <0.001 | 0.35 |
| Weight, mean (SD) | 86.38 (17.92) | 81.03 (16.56) | 75.12 (15.29) | 64.22 (9.88) | <0.001 | 0.83 |
| Height, mean (SD) | 171.56 (9.46) | 169.60 (9.18) | 165.83 (8.75) | 161.30 (10.07) | <0.001 | 0.61 |
| BMI, mean (SD) | 29.34 (5.69) | 28.05 (4.64) | 27.34 (5.38) | 24.85 (4.26) | <0.001 | 0.48 |
| Parent fractured hip, n (%) | 15 (7.5) | 9 (15.5) | 96 (22.7) | 4 (18.2) | <0.001 | 0.23 |
| Smoking, n (%) | 22 (11.1) | 6 (10.3) | 46 (10.8) | 4 (18.2) | 0.76 | 0.11 |
| Glucocorticoids, n (%) | 4 (2.0) | 2 (3.4) | 57 (13.5) | 1 (4.5) | <0.001 | 0.23 |
| Rheumatoid arthritis, n (%) | 7 (3.6) | 2 (3.4) | 18 (4.3) | 0 (0.0) | 0.77 | 0.15 |
| Secondary osteoporosis, n (%) | 43 (21.7) | 19 (32.8) | 131 (30.9) | 8 (36.4) | 0.08 | 0.17 |
| Alcohol, n (%) | 1 (0.5) | 1 (1.7) | 11 (2.6) | 0 (0.0) | 0.30 | 0.14 |
| Recent fracture, n (%) | 199 (100) | 58 (100) | 425 (100) | 23 (100) | 1.00 | 0 |
| ≥2 fx at recent fx event, n (%) | 45 (22.6) | 10 (17.2) | 68 (16.0) | 8 (34.8) | 0.05 | 0.24 |
| Months since fracture(s)*, mean (SD) | 2.00 (1.01) | 1.73 (0.85) | 2.03 (1.28) | 1.69 (0.69) | 0.18 | 0.22 |
| Older fracture**, n (%) | 65 (32.7) | 25 (43.1) | 202 (47.5) | 14 (60.9) | 0.002 | 0.31 |
| T-score femoral neck, mean (SD) | -0.91 (0.71) | -1.76 (0.43) | -2.22 (0.65) | -2.84 (1.13) | <0.001 | 1.36 |
| T-score total hip, mean (SD) | -0.14 (0.70) | -0.79 (0.56) | -1.36 (0.77) | -2.29 (0.92) | <0.001 | 1.54 |
| T-score lumbar spine, mean (SD) | -0.45 (1.24) | -1.19 (0.86) | -1.88 (1.12) | -3.54 (0.77) | <0.001 | 1.70 |
| Vertebral fracture (VFA), n (%) | 10 (5.0) | 5 (8.6) | 91 (21.4) | 21 (91.3) | <0.001 | 1.56 |
| FRAX***, mean (SD) | 10.61 (3.52) | 17.43 (6.15) | 27.00 (11.49) | 42.21 (19.05) | <0.001 | 1.56 |
| TBS, mean (SD) | 1.33 (0.13) | 1.32 (0.08) | 1.26 (0.10) | 1.20 (0.10) | <0.001 | 0.74 |
| Previous DXA examination, n (%) | 8 (4.0) | 5 (8.6) | 89 (20.9) | 1 (4.3) | <0.001 | 0.30 |
| Previous osteoporosis treatment, n (%) | 3 (1.5) | 1 (1.7) | 66 (15.5) | 0 (0.0) | <0.001 | 0.34 |
| Non Major osteoporotic Fracture, n (%) | 107 (53.8) | 23 (39.7) | 130 (30.6) | 7 (30.4) | <0.001 | 0.27 |

Baseline characteristics and comparisons per treatment. T-test and chi-square test were used. SMD = Standardized mean difference. SD = Standardized Difference. Fx = Fracture. TBS = Trabecular bone score. VFA=Vertebral fracture assessment. Older fracture=fracture that occurred prior to the FLS-fracture.

* Time between fracture date and examination date.

** Patients with a prior fracture before the fracture that qualified the patient for the FLS.

*** FRAX MOF 10-year probability.
